# T-follicular helper cells in malaria infection and roles in antibody induction

**DOI:** 10.1093/oxfimm/iqab008

**Published:** 2021-04-10

**Authors:** Megan S F Soon, Mayimuna Nalubega, Michelle J Boyle

**Affiliations:** 1 Department of Infectious Diseases, QIMR-Berghofer, 300 Herston Road, Herston, QLD, 4006, Australia; 2 Infectious Diseases Research Collaboration, Tororo District Hospital, Tororo, Uganda

**Keywords:** T-follicular helper, antibody, CD4 T cells, malaria, Vaccine

## Abstract

Immunity to malaria is mediated by antibodies that block parasite replication to limit parasite burden and prevent disease. Cytophilic antibodies have been consistently shown to be associated with protection, and recent work has improved our understanding of the direct and Fc-mediated mechanisms of protective antibodies. Antibodies also have important roles in vaccine-mediated immunity. Antibody induction is driven by the specialized CD4^+^ T cells, T-follicular helper (Tfh) cells, which function within the germinal centre to drive B-cell activation and antibody induction. In humans, circulating Tfh cells can be identified in peripheral blood and are differentiated into subsets that appear to have pathogen/vaccination-specific roles in antibody induction. Tfh cell responses are essential for protective immunity from *Plasmodium* infection in murine models of malaria. Our understanding of the activation of Tfh cells during human malaria infection and the importance of different Tfh cell subsets in antibody development is still emerging. This review will discuss our current knowledge of Tfh cell activation and development in malaria, and the potential avenues and pitfalls of targeting Tfh cells to improve malaria vaccines.

## INTRODUCTION

Despite gains in disease control over the last 20 years, malaria remains a disease of global importance responsible for ∼500 000 deaths and >200 million cases annually [1]. Alarmingly, reduction in malaria incidence has stagnated in the last 5 years, and the incidence is expected to rise following disruption to malaria control programmes due to the COVID-19 pandemic [2]. Malaria control programmes are hampered by the lack of a highly efficacious malaria vaccine. To date, the most advanced malaria vaccine, RTS,S, which targets sporozoite stage parasites that invade the liver, has only 55.8% efficacy in children (aged 5–17 months) and 31.3% efficacy in infants (aged ∼12 weeks) in the year following vaccination [3]. The World Health Organization has set the goal for the development of a malaria vaccine with >75% efficacy by 2030 [4]. To reach this goal, it is essential to improve our understanding of the mechanisms that mediate protection from malaria and identify strategies to target these mechanisms to improve vaccine efficacy in at-risk populations.

## ANTIBODIES AS KEY MEDIATORS OF NATURALLY ACQUIRED AND VACCINE-INDUCED IMMUNITY TO MALARIA

The key role of antibodies in protective immunity to malaria has been known since the 1960s. Seminal studies that transferred immunoglobulin (Ig) from malaria immune adults to children with malaria resulted in parasite clearance and disease resolution [5, 6]. While antibodies targeting malaria rarely induce sterilizing protection from infection, high levels of antibodies are thought to block parasite replication to prevent disease. Recently, gains have been made in our understanding of the specific mechanisms, types and functions of antibodies that mediate protection; these findings have been reviewed by a number of groups [7–10]. Cytophilic antibodies (IgG1 and IgG3 subclasses in humans, IgG2a/b in mice), which fix complement and bind Fc receptors (FcRs) to mediate cellular functions, are strongly associated with protection from malaria in human cohort studies. Data also support a role for malaria-specific IgM in protective immunity to malaria [11–18]. Cytophilic functional antibodies appear to be important in protection across all parasite stages. For example, complement-fixing antibodies against blood [19, 20] and hepatocyte stages are strongly associated with protection [21], and complement-fixing antibodies are also essential for blocking transmission stages within mosquitos [22–25]. Cytophilic antibodies are also important mediators of vaccine immunity induced by RTS,S [10, 26], whole sporozoite vaccine strategies [27–30], and blood-stage vaccines [31–33], and are associated with transmission-blocking activity induced by experimental transmission-blocking vaccines in humans [34].

## AGE AND MALARIA EXPOSURE MODULATE ANTIBODY INDUCTION

Compared to many other infectious diseases, antibody development to malaria is relatively slow, with individuals experiencing multiple parasite infections before protective antibody levels are achieved. As such, in naturally exposed populations, antimalarial antibodies increase with age and exposure and are consistently higher in adults and in populations in areas of high malaria transmission [8]. In these areas of high malaria transmission, age and exposure to malaria are co-linear, thus de-tangling the impact of each factor specifically is difficult in cohort studies. However, evidence suggests that age is an independent factor in antibody acquisition; modelling studies indicate that in high-transmission areas, antiparasitic immunity which is thought to be driven by antibodies, is driven by age and exposure independently [35]. Further, epidemiology studies of malaria naïve populations moving into malaria-endemic areas, show that adults acquire protection from symptomatic malaria infection and develop antimalarial antibodies more rapidly than children with the same malaria exposure histories [36, 37]. Age/exposure can also influence the types of antibodies that develop in response to infection [38–41]. For example, in naturally exposed populations, antibodies to merozoite surface protein 2 (MSP2) are polarized to IgG1 subclasses in infants and then switch to IgG3 dominant responses with increasing age [40, 41]. This switch in polarization to IgG3 is influenced by both age and exposure independently [39]. While increasing malaria exposure is associated with higher antibody levels, repeated malaria infection also drives changes to multiple aspects of the immune responses [42], which together may slow the acquisition of protective antibodies [43].

Age and exposure also influence the induction of vaccine-induced antibodies. Data from a Phase 3 trial showed that RTS,S vaccine efficacy was associated with age and exposure; efficacy was higher in vaccinated children aged 5–17 months compared to 6- to 12-week-old infants, and also was higher in low transmission compared to high transmission study sites [3]. These differences in efficacy are reflected by differences in vaccine-induced antibodies, with lower induced antibodies in infants compared to children, and reduced vaccine-induced antibodies in those with prior exposure [26, 44]. Induction of functional complement-fixing antibodies following RTS,S vaccination has also been shown to be age and exposure dependent in a Phase IIb RTS,S vaccine trial [45]. Age and exposure modulation of antibodies and vaccine efficacy have also been reported for whole sporozoite vaccines; the first study to assess *Plasmodium falciparum* sporozoite (PfSPZ) vaccine in various age groups reported higher antibody responses in Tanzanian infants compared to Tanzanian adults [46]. These infant antibody responses were comparable to those of malaria naïve US adults who received the same immunization regimen, consistent with hampered vaccine responsiveness due to prior malaria exposure [46]. Consistent with this hypothesis, comparison of responses between malaria-exposed adults to US malaria-naive adults showed whole sporozoite vaccination-induced lower antibodies and resulted in lower efficacy in exposed individuals who received the same immunization regimen [46–48]. Together data show that age/exposure impacts on antibody acquisition are likely important mediators of vaccine-induced efficacy. Understanding the specific mechanisms underlying age and exposure differences is important in identifying strategies to develop improved vaccines.

## T-FOLLICULAR HELPER CELLS AS THE CORNERSTONE OF ANTIBODY DEVELOPMENT

The discovery of T-follicular helper (Tfh) cells was first made in human tonsils when researchers noted a subset of CD4^+^ T cells with high expression of CXCR5, a B cell-zone homing marker [49, 50]. Tfh cells were found to be potent inducers of antibody production, a role once expected to be fulfilled by T-helper 2 (Th2) cells [51]. Tfh cells were eventually cemented as a distinct subset of CD4^+^ T cells, specialized in providing help to B cells, when Bcl6 was discovered as the lineage-defining factor [52–54]. Tfh cells are responsible for activating B cells within germinal centres (GCs) to generate high-affinity antibodies and memory B cell (MBC) responses including long-lived plasma cells which maintain circulating antibodies required for protection in multiple disease settings, including malaria. Due to the difficulty of studying Tfh cells in the context of the GC in humans, much of their development has been defined in mice [52–57]. The differentiation and regulation of Tfh cells have been extensively reviewed elsewhere [58–60].

While Tfh cells are conventionally defined by the expression of Bcl6, CXCR5 and PD-1, many other molecules have been described to mark Tfh cells including but not limited to ICOS, SAP, CD40 ligand, TCF-1 and PSGL1 [58]. Tfh cells also co-express markers of other CD4^+^ T cell lineages, often adopting characteristics of T-helper 1 (Th1), Th2, T-helper 17 (Th17) and T regulatory (Treg) cells. This finding was further emphasized when researchers detected the presence of circulating Tfh (cTfh) cells in human peripheral blood and noted that cTfh cells were present in distinct subsets, each with a unique phenotype and function which resemble the characteristics of the related CD4^+^ T-helper lineage [61–63]. Th1-cTfh cells (CXCR3^+^ CCR6^−^) express Tbet and produce IFNγ, while Th2-cTfh cells (CXCR3^−^ CCR6^−^) express GATA3 and produce Th2-related cytokines such as IL-4 and IL-5. Th17-cTfh cells express RORγt and produce IL-17, and commonly defined as CXCR3^−^ CCR6^+^. *In vitro* co-culture of naive B cells with subsets of cTfh cells have determined both Th2-cTfh and Th17-cTfh to be efficient helpers of naive B cells, inducing them to differentiate into Ig-producing plasmablasts, whereas Th1-cTfh cells lacked this capacity [61]. However, a separate study found that Th1-cTfh cells were the predominant cTfh subset detected following seasonal influenza vaccination, and Th1-cTfh cells were the most efficient subset to induce MBC differentiation [64]. Recently, Th13-Tfh/cTfh cells have been detected in both mice and humans to promote IgE production in response to allergens, emphasizing the highly heterogeneous nature of Tfh cells [65].

The relative importance of different Tfh cell subsets in antibody induction appears to be specific to the disease, pathogen or vaccine context. Th1-cTfh cells have been associated with antibody induction following infection with multiple viruses such as Human Immunodeficiency Virus (HIV) [66, 67], hepatitis C virus [68] and SARS-CoV-2 [69, 70], as well as vaccination to influenza, which predominantly boosts memory responses [64, 71]. Both Th2-cTfh and Th17-cTfh cells were positively correlated with the frequency of blood plasmablasts in autoimmune diseases [61, 72]. Th2-cTfh cells have also been implicated with antibody production in malaria [73], while antibody responses in Ebola and HIV were correlated with Th17-cTfh cells [74, 75]. More work is required to precisely elucidate the functional relevance of each Tfh subset in various disease settings. It is currently unclear how Tfh cells differentiate into such highly heterogeneous states. However, data have suggested that specific factors can be targeted to manipulate Tfh subset development, with a recent study in systemic lupus erythematosus identifying ETS1 as a specific repressor of Th2-Tfh cells [76].

Along with heterogeneous subset differentiation, recent seminal studies in mice have employed adoptive transfer and longitudinal cellular tracking to clearly establish that Tfh cells generate memory and are linearly derived from their effector progenitors [56, 77–79]. An important observation noted by these studies was that memory Tfh cells tend to downregulate many Tfh-associated molecules. CXCR5 appears to be the rare exception and is maintained in the memory state and is therefore the most reliable marker known thus far to identify memory Tfh cells. Nevertheless, the advent of technological advances of transcriptomic and epigenomic approaches are improving our resolution for studying memory Tfh cells [77, 78]. Using the microarray technique, folate receptor 4, a receptor for folic acid which is often associated with CD4^+^ Treg cells [80], was surprisingly discovered to be upregulated in murine Tfh cells and was retained in memory Tfh cells, albeit at a lower level [78, 81]. Differential methylation patterns on the *Gzmb* (encoding for Granzyme B) locus were also found to contribute in preserving the lineage commitment in memory Tfh versus Th1 cells [77]. In humans, researchers have classified cTfh cells as both memory [61, 62, 64, 82] and recently-activated GC Tfh cells that have exited lymphoid tissues into the circulation [73, 83]. Recently, elegant studies have confirmed that blood cTfh are directly related to Tfh cells within lymph nodes and circulate via thoracic duct lymph [84]. Importantly, ICOS^+^ CD38^+^ cTfh cells were enriched for GC Tfh transcriptional and epigenetic signatures, highlighting that activated Tfh in the periphery expressing ICOS and CD38 likely represent recently egressed Tfh cells [84]. These findings are supported by other T cell receptor (TCR) clonal analyses reporting significant clonal overlap between cTfh cells and tonsillar GC Tfh populations [83, 85]. Perhaps the most consistent idea from these studies is that Tfh cells can traffic in and out lymphoid sites, both during an active GC reaction and also as a circulating pool of memory cells. This concept has been confirmed in studies of seasonal influenza vaccine where cTfh have been shown to be reactivated following vaccination with memory populations cycling between ICOS^+^ CD38^+^ (activated) and ICOS^−^ CD38^−^ (resting) states [82]. There remain large gaps in our understanding of the development and heterogeneity of Tfh cells, the role of Tfh cell subsets in antibody induction in different disease contexts and the development and role of Tfh memory cells in protection from diseases. However, data hint that Tfh cells exist in a plastic state where they can be malleable to the type of stimuli they are exposed to, making them extremely useful tools for skewing antibody production in a targeted manner.

## LESSONS LEARNT FROM MOUSE STUDIES OF MALARIA

In recent years, researchers have utilized experimental malaria models to delineate the factors crucial for Tfh responses during malaria. Several mouse models have been used to explore GC reactions during blood-stage infection, including non-lethal *Plasmodium chabaudi chabaudi* AS (*P. chabaudi*), non-lethal *P. yoelii* 17XNL (*P. yoelii*) and *P. berghei* ANKA (*P. berghei*). Of these, both *P. chabaudi* and *P. yoelli* are most widely used to study antibody-dependent mechanisms during malaria since B cell responses were shown to be crucial for either the complete resolution of parasitaemia or the survival of mice in these two models [86–88]. On the other hand, *P. berghei* induces a lethal infection that cannot be controlled by antibodies in wild-type mice [89]. It is also difficult to study GC reactions in *P. berghei-*infected mice as they would normally succumb to the infection before GC responses could occur, unless intervened with cures such as antimalarial drugs [90]. Here, we will focus on reconciling data from studies employing *P. chabaudi* and *P. yoelli* to understand Tfh and subsequent GC B cell responses and antibody induction.

Although Bcl6 has been recognized to be the lineage-determining transcription factor for Tfh cells in viral infection as early as 2009 [52], it has only recently been determined that CD4^+^ T cell-intrinsic Bcl6 signalling is also required for the induction of Tfh responses in malaria [91]. Deletion of Bcl6 in CD4^+^ T cells abrogated Tfh (CXCR5^+^ PD-1^+^ and CXCR5^+^ Bcl6^+^) responses, and resulted in reduced numbers of GC B cells in the *P. chabaudi* model. Although IgM induction was not affected, the production of all parasite-specific IgG responses (IgG1, IgG2b, IgG2c, IgG3) failed in the absence of Bcl6-expressing CD4^+^ T cells. While Bcl6 is required for both the generation and differentiation of Tfh cells, ICOS only comes into play later when it helps promote the interaction of Tfh and B cells at the GC to maintain long-term Tfh cell responses [92]. Indeed, early Tfh cell differentiation was not impacted by the absence of ICOS during *P. chabaudi* infection. Other co-stimulatory molecules are also essential in Tfh cell development in malaria, with complex cross-talk between different co-stimulatory and co-inhibitory molecules [88, 93]. For example, using the *P. yoelii* model, Tfh cell responses were augmented by combinatorial blockade of PD-L1 and Lag3 [88], but abrogated by the coordinate ligation of OX40 and PD-1 blockade [93]. In agreement to Bcl6’s essential role for Tfh cell responses in *P. chabaudi* [91], the downregulation of Bcl6 in Tfh cells due to combined stimulation of OX40 and PD-1 blockade also led to an impaired GC response during *P. yoelii* infection [93]. Importantly, all these studies demonstrated a correlation between Tfh cells and GC B cell and antibody responses. When Tfh cell responses were improved, parasite-specific IgG titres increased. In some instances, passive transfer of serum from mice with better Tfh cell responses also conferred better protection [88, 92].

Tfh cell development during malaria is also modulated by cytokines. Generation of Tfh cells in mice depends on IL-6 and IL-21 [94, 95]. However, it is important to note that IL-6 and IL-21 work redundantly and only the combined absence of both cytokines suppress Tfh cell differentiation. In murine malaria, both of these cytokines were similarly shown to support various aspects of GC development [96, 97]. GC B cell responses, antibody-secreting cells and production of parasite-specific antibodies were all reduced in IL-21^−/−^ mice during *P. chabaudi* infection [96]. Interestingly, Tfh cell responses were normal in these mice and it was speculated to be due to compensatory effects by IL-6. This highlights that IL-21 signalling in B cells is also crucial for optimal humoral immunity in malaria, in agreement with previous reports demonstrating the role of B cell-intrinsic IL-21 for plasma cell differentiation *in vitro* and GC B cell proliferation *in vivo* [98, 99]. In the absence of IL-6, GC B cell responses were disrupted in both the *P. chabaudi* and *P. yoelii* models [97]. Accordingly, parasite-specific antibodies were also reduced in IL-6^−/−^ mice. The lack of IL-6 only modestly affected Tfh cells by reducing the levels of ICOS expression in both malaria models. Collectively, data from these two reports support the idea that IL-6 and IL-21 play redundant roles in supporting Tfh cells during malaria, but are both independently important for supporting subsequent GC B cell responses. However, it is unknown how these findings in mice can be extrapolated to human infection, as IL-6 appears not to be required for induction of Tfh cells in humans. IL-12 has instead been implicated to induce expression of various Tfh markers in human CD4^+^ T cells [100, 101]. *In vitro* differentiation of human Tfh cells can be achieved by culturing CD4^+^ T cells in the presence of IL-12 and TGFβ or activin-A, but the same conditions failed to induce murine Tfh cells *in vitro* [101, 102].

Another cytokine signalling pathway that has been gaining traction for regulating Tfh cell responses in malaria is the Type I interferon (IFN) signalling pathway. Several groups have now reported that Tfh cells and Tfh-mediated GC responses were compromised by Type I IFN signalling during malaria, in both the *P. yoelli* and *P. chabaudi* models [103, 104]. Sebina *et al.* illustrated that the effects of Type I IFN signalling on Tfh cells proceeded via conventional dendritic cells to limit ICOS expression on Tfh cells, leading to decreased GC B cell responses, parasite-specific antibodies and control of parasite growth [103]. Interestingly, the Tfh cell-suppressive nature of Type I IFN signalling observed by the Butler group was determined to be due to Type 1 regulatory (IFNγ/IL-10 producing T cells; Tr1)-intrinsic Type I IFN signalling [104], indicating that there may be multiple mechanisms in how Type I IFN signalling modulates Tfh cell responses in malaria. Interferon regulatory factor 3 (IRF3), one of the upstream molecules of Type I IFN signalling pathway was also shown to suppress Tfh cells [105]. Using a competitive mixed bone marrow chimera approach, IRF3 was revealed to act in B cells to limit GC B cell responses which then obstructed support for Tfh cells. In the absence of IRF3, parasite-specific antibodies (IgM, IgG1, IgG2b, IgG2c, IgG3) were increased in both the *P. chabaudi* and *P. yoelii* models, again correlating improved Tfh cell responses with better humoral immunity in malaria.

An important question raised by these studies is the relationship between IFNγ and Tfh cells. IFNγ has been traditionally defined as a classical Th1 cytokine and hence often thought to be antagonistic to Tfh cells, however recent data suggest that this relationship may be more complex. First, Tfh cells have been observed to produce IFNγ alongside IL-21 during murine malaria [106, 107]. This suggests heterogeneity or plasticity in Tfh cells generated during *Plasmodium* infection, in line with the observations in human malaria patients [108]. More importantly, various reports illustrated that IFNγ blockade alone only resulted in modest changes to Tfh cells in malaria. In an attempt to enhance Tfh cell functionality, Zander *et al.* discovered that IFNγ and IL-10 cooperate to impede Tfh cell, GC B cell and antibody responses, however Tfh cell suppression by either cytokine were minimal when examined independently [104]. In a separate study, the authors also observed that administration of OX40 agonist alone promoted Th1 cell responses alongside Tfh cells, highlighting that increased levels of IFNγ may not always be detrimental [93]. It was only during excessive IFNγ production due to combined OX40 ligation and PD-1 blockade that Tfh cell responses were impaired. This was also mirrored in a number of other studies where Tfh cell responses were either unaffected or could still be boosted simultaneously as improved Th1 cell responses were seen [92, 103]. In a study with *P. berghei*, pro-inflammatory conditions during severe malaria stunted Tfh cell development by inducing expression of Tbet and CXCR3 on Tfh cells [90]. Interestingly, when the authors tried to lift the suppression of Tfh cells by administering anti-IFNγ blocking antibody alone, the effect was minimal. The differentiation of Tfh cells was only restored with the co-blockade of IFNγ and TNFα. Therefore, these data remain consistent with the idea that Th1 or IFNγ responses can limit Tfh cells in malaria, however also serve to emphasize that the levels of IFNγ and/or co-operation with other molecules will need to be taken into context. Indeed, others have suggested that the Th1 and Tfh cells can exist as a continuum, where the balance between Bcl6, STAT3, Blimp-1 and Tbet expression in CD4^+^ T cells determine the predominant Th subset in effector CD4^+^ T cells in malaria [106, 109]. However, studies using single-cell RNA sequencing (scRNAseq) showed that transgenic *Plasmodium*-specific CD4^+^ T cells bifurcated into two separate lineages, Th1 and Tfh *in vivo* [110]. We also recently extended on that study to demonstrate that the transition of these *Plasmodium*-specific CD4^+^ T cells from Th1 and Tfh effector cells to lineage-committed memory cells occurred with minimal lineage plasticity *in vivo* [111]. Nevertheless, the expression of certain classical Th1 and Tfh molecules were indeed shared between the two cell fates in both studies (e.g. *Ifng*, *Tbx21*, *Cxcr3*) [110, 111]. Interestingly, there was substantial transcriptomic and epigenomic coalescence between the two memory lineages during the transition from effector to memory, but the lineage distinction between memory Th1 and Tfh cells could be considerably improved by allowing memory cells to develop in the absence of a persisting *Plasmodium* infection [111]. Additionally, the functional quality of these memory cells was also enhanced when developed in a non-persistent infection. While memory Tfh-specific recall responses were not assessed here, this work nonetheless revealed that memory Tfh cells arise from effector Tfh cell precursors in malaria. Both Th1-like and Tfh-like memory cells in mice have been reported to recall Tfh cell functions upon secondary challenge of *Plasmodium*, implying that memory CD4^+^ T cells exhibit functional overlap during recall responses [112]. It remains unknown what level of lineage persistence is retained in memory Tfh cells upon re-infection, with limited reports hinting at the multipotency of memory Tfh cells in other infection models [113].

Other more global factors may also be important in Tfh cell development in malaria. Recently, changes in cellular metabolism and microbiome composition have been recognized to be important factors in influencing immune responses across many disease settings. *Plasmodium*-specific CD4^+^ T cells were noted to undergo dramatic changes in their cellular metabolism as they differentiated into effector Th1 and Tfh cells [110]. Of note, several metabolic-related molecules such as *P2rx7* (encoding P2X7) and *Entpd1* (encoding CD39) were found to correlate with Th1 and Tfh cell bifurcation in malaria. Subsequently, it was shown that P2X7, a receptor for extracellular ATP, was indeed activated in CD4^+^ T cells during malaria following the rupture of infected erythrocytes [114]. P2X7 activation favoured Th1 over Tfh cells and the deletion of P2X7 improved levels of serum IL-21, GC reactions, as well as parasite-specific Ig levels, indicating a role for P2X7 in fine-tuning the balance between Th1 and Tfh cells, both of which are required for optimal control of *Plasmodium* parasites. Exacerbated production of glucocorticoids due to sleep deprivation has also been recently revealed to compromise Tfh cells and subsequent antibody responses during *P. yoelii* infection [115]. Additionally, gut microbiota can affect a range of physiologic functions, including CD4^+^ T helper cells [116]. One such example is seen in how gut commensal segmented filamentous bacteria drove differentiation and egress of Tfh cells from the Peyer’s patches into systemic lymphoid sites, leading to exacerbated GC and autoantibody responses responsible for gut-distal autoimmune arthritis [117]. To date, there are only limited studies of the role of gut microbiota in CD4^+^ T cell responses in malaria. Villarino *et al.* first showed that differences in gut microbiota composition determined the severity of malaria in mice, and that disease resistance was associated with an elevated humoral response, providing the first hint between Tfh cells and gut microbiota in malaria [118]. This was then confirmed by the same group recently, where gut microbiota was shown to temper with various GC responses, including Tfh cells, plasmablasts and GC B cells during malaria [119]. While the exact mechanisms for how gut microbiota act to control Tfh cells and related GC responses remain to be determined, an interesting finding from this study was that the B cell receptor repertoires of malaria-resistant mice were more diverse than those of the susceptible mice, suggesting that GC processes such as somatic hypermutation and the breadth of circulating *Plasmodium*-specific antibodies can be modulated by gut microbiota composition.

## LESSON LEARNT FROM HUMAN STUDIES OF MALARIA

While the central role of Tfh cells in antibody development in malaria has been clearly demonstrated in animal models, to date relatively few studies have specifically investigated Tfh cells during human malaria. In Malian children (age 6–12), *P. falciparum* malaria robustly activated cTfh cells, with increased expression of ICOS, HLA-DR, CD38 and Ki67 detected within cTfh cells during malaria compared to post-treatment [108]. However, cTfh cell activation was restricted to Th1-cTfh subsets (CXCR3^+^) both *in vivo* during malaria, and *in vitro* following *P. falciparum* parasite stimulation of peripheral blood mononuclear cells (PBMCs) from exposed children [108]. As shown in malaria-naive adults [61], Th1-cTfh cells in malaria-exposed children had reduced capacity to activate naïve B cells compared to Th2/Th17-cTfh subsets [108]. Indeed, while antimalaria IgG responses increased in Malian children after a malaria episode, the increase in breath and magnitude of the antibody response was not associated with Th1-cTfh cell activation. Instead, the inflammatory IFNγ cytokine milieu during malaria and Th1-cTfh cells activated by parasite simulation have been implicated in the induction of Tbet^+^ ‘atypical’ MBC responses [120], which have been previously thought to have exhausted phenotypes and contribute to slow acquisition of immunity [121–124]. Consistent with these findings, is our recent data linking Th2-cTfh cell activation with functional antibody development during experimental *P. falciparum* blood-stage infection in Australian adults [73]. Within this experimental infection model, Th2- (CXCR3^−^ CCR6^−^) and Th1-cTfh (CXCR3^+^CCR6^−^) subsets were activated with different kinetics. Th2-cTfh cell activation peaked 8 days after inoculation (time of parasite treatment), and then Th1-cTfh cells were activated after parasite treatment by Day 15. Importantly, only Th2-cTfh cell activation was associated with the induction of functional antimalarial antibodies. Furthermore, while atypical MBC responses were not investigated in this cohort, Th1-cTfh cell activation was associated with plasma cell induction following treatment [73]. The majority of these induced plasma cells are thought to be short-lived and have been shown to act as a nutrient sink to the detriment of GC B cell activation and MBC development [125]. Data are supported by a previous study of *P. vivax* malaria in Brazilian adults which showed a positive association between Th2-cTfh cells and antimalarial antibodies which was strongest in patients with multiple prior malaria infections [126]. Together data support a positive role of Th2-cTfh cell activation in antibody development in malaria, and suggest that Th1-cTfh cell responses may hamper immunity via multiple immunoregulatory pathways.

However, other data suggest multiple potential supportive roles of Th1-Tfh subsets in antibody development and induction of protective immunity. While ‘atypical’ MBCs have been thought of as detrimental to the development of protective immunity [121–124], recent data have shown that ‘atypical’ MBCs can mount a rapid recall response and give rise to antibody-producing cells, implicating a role for atypical MBCs in protection [127]. Further, atypical MBCs have the highest levels of IgG3 expression of all MBC subsets [120]. IgG3 antibodies have the highest capacity to fix complement and are consistently associated with protection from malaria in human cohort studies, as such the activation of atypical MBCs by Th1-Tfh may be essential in the development of cytophilic and protective responses. Consistent with this hypothesis, IFNγ is responsible for isotype switching to cytophilic subclasses (IgG2a/c in mice and IgG1/3 in humans) [128, 129]. Indeed, recent studies in ehrlichial murine models have shown that IL-21 and IFNγ from Th1-Tfh cells were specifically required to induce Tbet^+^ B cells and class switching to cytophilic IgG2c [130] and Tbet expression in B cells is required for IgG2a/c switching in viral infection [131]. Moreover, while Th1-cTfh cells have inferior capacity to activate naïve B cells, they have higher capacity to activate MBCs *in vitro* [64] and are consistently associated with the activation of MBC responses following influenza vaccination [64, 71]. As such, Th1-Tfh may become more important in re-activation of memory responses as immunity develops.

Due to the paucity of studies on Tfh cells during human malaria, our understanding of specific mechanisms of Tfh cell development, the impact of age and exposure, Tfh cell memory and recall responses, and links to antibody production remains limited. Type I IFN signalling has been shown to be an important driver of Tr1 cell responses in human malaria infection [132] and parasite-specific Tr1 cells dominate CD4^+^ T cell responses in malaria [133–135]. However, to date, no studies have investigated the impact of Tr1 cells on Tfh cell development during malaria in humans, despite an important agonistic role of IFNγ and IL-10 on Tfh during murine *Plasmodium* infection [104]. Further, there is limited data regarding the longitudinal development of Tfh cell subsets and Tfh cell memory. Within *P. vivax* malaria-infected adults, the total magnitude of activated cTfh increased with the number of prior malaria episodes suggesting prior exposure boosts existing memory Tfh cell responses. However, subset distribution of activated cTfh cells was not reported across different exposure histories [126], and it remains unknown whether the subsets distribution of cTfh cells changes with repeated malaria infection. Of particular note, to date, all studies of Tfh in human malaria have relied on peripheral blood sampling, and none has investigated Tfh cells within GCs directly. The use of peripheral samples to understand human responses to infection and vaccination has been extensively used in the field, largely due to the difficulty in obtaining samples from lymphoid tissue. Despite the utility of peripheral blood sampling, studies that take advantage of sampling from lymphoid tissues, such as via ultra-sound guided fine needle aspiration [136], are powerful approaches to revolutionize our understanding of antibody development. For example, recent studies of GC B cells in humans collected via serial sampling with fine-needle aspiration following influenza vaccination, have shown that vaccination induces recall memory responses which are cross-reactive to multiple influenza strains, and also activates naïve B cells which predominantly encode strain-specific antibodies [137]. GCs within the spleen are also essential sites of immune activation of malaria with the spleen considered the main lymphoid organ involved in the development of immunity to malaria, and also independently as an important site for clearance of blood-stage parasites [138]. However, malaria causes disruption of splenic architecture in both murine [90, 139–142] and non-human primate (NHP) models [143], and in humans [144]. Murine studies have suggested that these changes to splenic architecture may only have minimal impact on parasite-induced [140] or vaccine-driven antibody responses [139]. However, there are multiple important differences between mice and human spleens [145] and it is possible that malaria-driven disruption to splenic architecture negatively impacts antibody development in humans. Investigating these issues will require creative studies that overcome multiple logistical challenges in acquiring splenic samples from malaria exposed and malaria-infected individuals. Alternatively, the use of recently developed methods to generate lymphoid organoids *in vitro* may provide insights regarding the impact of *Plasmodium* parasites on human GC and antibody development [146].

## TARGETING Tfh CELLS BY VACCINATION TO IMPROVE EFFICACY

Due to the central importance of Tfh cells in antibody development, targeting this CD4^+^ T cell subset has been proposed as an avenue to improve vaccine efficacy [147], including in malaria [7]. Encouragingly, despite our limited understanding of Tfh cells in human malaria infection and vaccination, recent studies have suggested that optimized Tfh cell activation by malaria vaccines may indeed improve efficacy in humans. In a Phase 1b clinical trial of an experimental malaria vaccine targeting *P. falciparum* trophozoite exported protein 1 antigen, vaccine adjuvanted with the TLR4 agonist glucopyranosyl lipid adjuvant-stable emulsion (GLA-SE) induced higher cTfh cell responses compared to alhydrogel (Alum) adjuvant vaccination [148]. Increased cTfh cell activation by the GLA-SE vaccine was associated with increased magnitude and longevity of induced antibodies. No differences in the Th1/Th2/Th17 composition of the activated cTfh cells were detected between the two vaccines, indicating that boosting the magnitude of the Tfh cell response may be sufficient in increasing vaccine efficacy in some cases. Other adjuvants have been shown to boost the magnitude of Tfh cells and improve antibody development. For example, the emulsion-based adjuvant MF59 boosts Tfh cell induction and GC formation in NHP vaccination [149], and CpG-DNA adjuvant increases Tfh cell and antibody responses to influenza vaccination, compared to responses to un-adjuvanted vaccines in humans [150]. Other vaccine delivery platforms such as nanoparticles, may also be beneficial, and have been shown to boost Tfh cells and antibody induction to malaria antigens in mice [151], and NHP models [152]. Similarly, mRNA vaccines encapsulated in lipid nanoparticles induce high levels of Tfh cells and potent antibodies in mice and NHPs [153].

More crucially, boosting specific Tfh cell subsets that are involved in antimalarial antibody induction may be important. Studies have shown that co-administration of RTS,S vaccine (adjuvanted with AS01B a liposome-based vaccine adjuvant system containing 3-O-desacyl-4'-monophosphoryl lipid A and saponin QS-21 which acts as a TLR4 agonist), with a viral vectored vaccine platform consisting of chimpanzee adenovirus serotype 63 and modified vaccinia virus Ankara, resulted in a Th1-cTfh cell bias, lower antibody induction, reduced inhibition of parasites and decreased vaccine efficacy in humans [154]. This finding is consistent with our data linking Th2-cTfh cell activation with antimalarial antibody development [73] and also supported by a recent observation that Th2-cTfh skewed responses correlated with improved antibody production when the malaria vaccine candidate antigen PfRH5 was delivered using protein adjuvanted with AS01B [155]. These findings highlight the need for a clear understanding of how specific Tfh cell subsets induce antimalaria antibodies in humans. Attempts to boost Th1-Tfh cells have been made in HIV vaccines in NHP models, with the addition of the Th1-chemokine interferon-induced protein-10 (a CXCR3 ligand and inducer) to DNA vaccine regimes resulting in increased Th1-signatures within GC Tfh cells and enhanced GC B cell responses and induced antibodies [156]. Development of similar approaches for other Tfh cell subsets will require improved understanding of specific mechanisms for driving Tfh cell subset development and responsiveness to vaccination.

Along with specific Tfh cell targeting adjuvants, other strategies may also improve Tfh cell induction by vaccination. For example, delayed fractional dosing of RTS,S vaccine improved vaccine efficacy in some cases [157, 158], and improved efficacy of Tfh cell responses have recently been implicated in fractional dose regime [159]. While the specific mechanisms underpinning improved efficacy in delayed fractional dosing are unknown, antigen concentrations and availability may be important [160]. Optimized spacing between vaccine doses has also been shown to enhance GC Tfh cell and B cell responses in HIV vaccines in NHP models [161, 162]. Additionally, the mode of vaccine delivery may also impact Tfh cell activation, with subcutaneous vaccination of a nanoparticle in NHP models inducing 10-fold higher Tfh cell and B cell response within draining lymph node GCs compared to intramuscular vaccination [163].

## APPLYING Tfh TARGETING VACCINES TO AT-RISK POPULATIONS

While multiple avenues for improving Tfh cell responses in vaccination have been identified, applying these strategies to at-risk populations may be met with additional challenges. Tfh cell responsiveness to vaccination is known to be reduced in aged populations [164, 165], but to date Tfh cell responsiveness in infants and children compared to adults has only been studied in limited settings. Age-driven increases to the frequency of cTfh cells have been identified in malaria-exposed children and associated with RTS, S vaccine antibody induction [166]. In mice, neonates have reduced Tfh cell induction following OVA-alum immunization compared to adults [167], and preferentially differentiated into short-lived pre-Tfh effector cells in responses to tetanus-alum vaccination [168]. Both environmental and T cell-intrinsic factors appear to influence these age-dependent changes to Tfh cell responsiveness [169]. However, specific adjuvants appear to overcome these age-dependent responses to vaccination, with CpG [168, 169], R848 [170], CAF01 [171] and MF59 [172, 173] adjuvant vaccines all enhancing neonatal responses to adult levels in animal models. In contrast, in some models of vaccination, neonatal Tfh cell responses appear to be higher than adults, e.g. HIV-envelope immunization in macaques [174]. Indeed, antibody induction in humans following MF59-adjuvanted HIV envelope vaccines is higher in infants compared to adults [175]. Similarly, vaccination with live-attenuated influenza vaccines in humans results in higher Tfh cell responses, and antibody induction in children compared to adults [176]. To rationally design Tfh-targeting malaria vaccines for infant and children populations, improved understanding of the mechanisms underpinning different Tfh cell responses to specific vaccines across age groups is needed.

RTS,S vaccine efficacy is reduced in areas of high malaria transmission, suggesting that malaria-driven changes to immune systems may be important. In Malian children, Tfh cell activation following parasite stimulation of PBMCs is restricted to Th1-cTfh subsets [108], in contrast to malaria-naive adults where all cTfh cell subsets are activated by parasite stimulation *in vitro* [73]. This may suggest that malaria exposure drives changes to the ability of Tfh cells to respond to stimulation, however, whether this impacts responsiveness to vaccination is unknown. Similarly, malaria drives parasite-specific Tr1 cells, which dominate the response in malaria-exposed children [133–135]. Given that Tr1-derived IFNγ and IL-10 cytokines negatively regulated Tfh cell responses in mice models of malaria [104], overcoming or blocking parasite-specific Tr1 cells in malaria-exposed children may be required to optimize vaccine responsiveness. Host-directed therapies or immune check point blockade may be one such approach to overcome malaria-driven CD4^+^ T cell imprinting. This concept has been explored in *P. yoelii*-infected mice, where blockade of PDL-1, LAG-3 and CTLA-4 enhanced Tfh cell responses and production of functional antibodies by B cells resulting in rapid control of parasite burden and accelerated parasite clearance [88, 177]. Given the heterogeneity human Tr1 responses, blocking a single target may only offer small modulation of immune responses. However, Type I IFN signalling has been identified as key mediators of Tr1responses in both rodent and human malaria [103, 104, 132, 178, 179], and targeting Type I IFN signalling can improve effector T cell responses in animal models of other parasitic infections [180]. Whether such approaches can boost malaria responsiveness to vaccination is unknown [181]. Along with malaria-driven changes to Tfh and CD4^+^ T cells, other confounding factors may also need consideration in Tfh cell-targeted therapies. For example, anaemia has been linked to RTS, S responsiveness in children [166] and low serum iron impairs plasmablast generation *in vitro* and responsiveness to vaccination including Tfh cell activation in animal models [182]. Together, data highlight that multiple challenges remain in designing Tfh cell-targeted vaccine platforms for malaria in at-risk populations.

## CONCLUSIONS AND FUTURE DIRECTIONS

**Figure 1:**
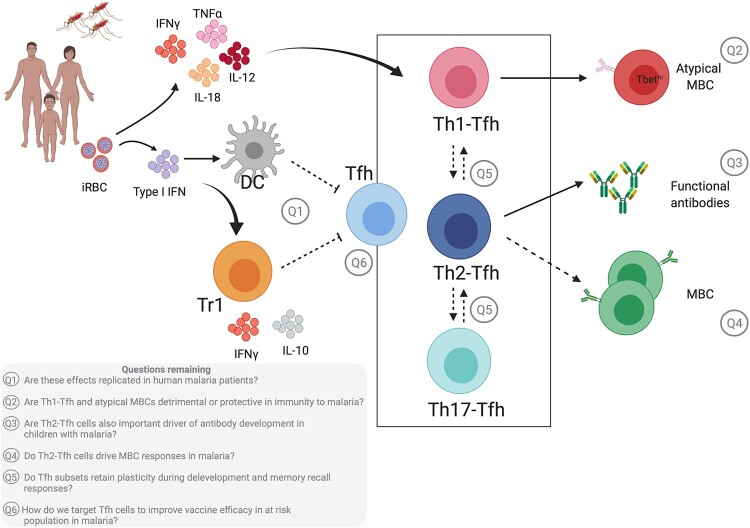
Tfh cells in antibody malaria development in humans and key questions. Subsets of Tfh cells (Th1-cTfh, Th2-cTfh and Th17-cTfh) are present in clinical and experimental malaria settings. In high-transmission settings, *Plasmodium* infection in children induces pro-inflammatory cytokine production (IFNγ, TNFα, IL-18, IL-12) which drives Th1-cTfh responses [108, 120]. *Plasmodium* infection in humans and mice activates Type I interferon (IFN) signalling [103, 104, 132], which signals through murine dendritic cells (DCs) [103] or Type 1 regulatory (Tr1) cells [104] to suppress Tfh and subsequent antibody responses. It remains unknown whether Type I IFN signalling via DCs and Tr1 cells are important negative regulators of Tfh development during human infection (Q1). Preferential activation of Th1-cTfh cells in children from malaria-endemic regions is associated with atypical MBC responses [108, 120]. However, it is unclear whether Th1-Tfh and atypical MBCs have positive or negative roles in protective immunity (Q2). We have shown that Th2-cTfh cell activation in malaria-naïve adults undergoing volunteer infection with *Plasmodium* parasites is positively correlated with high levels of functional antibody responses [73]. Whether Th2-Tfh cells also drive functional antibody development in children during natural exposure (Q3), and their role in MBC development is unknown (Q4). Also, it is not yet known if Tfh subsets retain plasticity after differentiation and during memory recall responses (Q5). Together, while Tfh cells are an attractive target to boost vaccine efficacy, specific knowledge on how to best boost Tfh to improve vaccine antibody induction is unknown, and translation of these approaches to the field may face additional challenges (Q6). Solid line: data shown in humans; Dotted line: data not yet shown in humans. iRBC—infected red blood cells. Key gaps in the field are highlighted in the grey box. Image created using BioRender.com.

As the cornerstone of antibody development, Tfh cells are attractive targets for improving vaccine efficacy. In order to do so for malaria vaccines, it is essential that we improve our understanding of the mechanism of Tfh cell biology and function in malaria infection and vaccination. While much has been learnt from animal models, further studies are required in humans to inform the development of Tfh cell target vaccines (Fig. 1). Specifically, it is essential that we strengthen our understanding of the factors mediating Tfh cell activation in malaria, the role of specific Tfh cell subsets in antibody induction and longevity in children, and how age and malaria exposure impact Tfh cell development and responsiveness to vaccination. Specific longitudinal studies of individuals with multiple rounds of malaria infection are required to understand the development and plasticity of Tfh cell subsets with repeat infections and roles of specific Tfh cell subsets in activating naïve and MBCs during malaria infection. Although large key knowledge gaps remain, encouraging results investigating links between Tfh cell boosting vaccines strategies and antibody development in human malaria vaccines, highlight the potential of Tfh cell targeting and provide encouragement for future vaccine development.

## Conflict of interest statement

No conflicts to declare.

## DATA AVAILABILITY STATEMENT

There is no data associated with this review article.
